# Thai lexical tone perception in native speakers of Thai, English and Mandarin Chinese: An event-related potentials training study

**DOI:** 10.1186/1471-2202-9-53

**Published:** 2008-06-23

**Authors:** Edith Kaan, Christopher M Barkley, Mingzhen Bao, Ratree Wayland

**Affiliations:** 1 Linguistics, University of Florida, Box 115454, Gainesville, FL 32611, USA; 2 Department of Linguistics, University of California at San Diego, 9500 Gillman Drive #108, La Jolla, CA 92093, USA

## Abstract

**Background:**

Tone languages such as Thai and Mandarin Chinese use differences in fundamental frequency (F_0_, pitch) to distinguish lexical meaning. Previous behavioral studies have shown that native speakers of a non-tone language have difficulty discriminating among tone contrasts and are sensitive to different F_0 _dimensions than speakers of a tone language. The aim of the present ERP study was to investigate the effect of language background and training on the non-attentive processing of lexical tones. EEG was recorded from 12 adult native speakers of Mandarin Chinese, 12 native speakers of American English, and 11 Thai speakers while they were watching a movie and were presented with multiple tokens of low-falling, mid-level and high-rising Thai lexical tones. High-rising or low-falling tokens were presented as deviants among mid-level standard tokens, and vice versa. EEG data and data from a behavioral discrimination task were collected before and after a two-day perceptual categorization training task.

**Results:**

Behavioral discrimination improved after training in both the Chinese and the English groups. Low-falling tone deviants versus standards elicited a mismatch negativity (MMN) in all language groups. Before, but not after training, the English speakers showed a larger MMN compared to the Chinese, even though English speakers performed worst in the behavioral tasks. The MMN was followed by a late negativity, which became smaller with improved discrimination. The High-rising deviants versus standards elicited a late negativity, which was left-lateralized only in the English and Chinese groups.

**Conclusion:**

Results showed that native speakers of English, Chinese and Thai recruited largely similar mechanisms when non-attentively processing Thai lexical tones. However, native Thai speakers differed from the Chinese and English speakers with respect to the processing of late F_0 _contour differences (high-rising versus mid-level tones). In addition, native speakers of a non-tone language (English) were initially more sensitive to F_0 _onset differences (low-falling versus mid-level contrast), which was suppressed as a result of training. This result converges with results from previous behavioral studies and supports the view that attentive as well as non-attentive processing of F_0 _contrasts is affected by language background, but is malleable even in adult learners.

## Background

Variation in voice pitch, an auditory impression of the rate of vocal fold vibration (F_0_), plays a different linguistic function in tone and non-tone languages. Tone languages, such as Thai and Mandarin Chinese, use differences in either average F_0 _or F_0 _contours (or slopes) over strings of otherwise identical phonemes to distinguish between different words in the lexicon from one another. For instance, the Thai syllable [k^h^a:] means something completely different when pronounced with a tone that is low-falling ("galangal root"), low-falling and then rising ("leg"), high-falling ("I, servant"), high-rising ("to do business in") or mid-level ("to be lodged in"). In non-tone languages such as English, on the other hand, pitch variation is not used to differentiate word meaning. However, even though F_0 _is not used to distinguish meaning between words in English, it can make one syllable more perceptually prominent or more salient than neighboring syllables in multi-syllabic words. For example, the first syllable of the word 'cookie' is stressed, and perceptually more salient than the second syllable. The F_0 _or pitch (as well as intensity or loudness and vowel duration) of the stressed syllable is typically higher than its neighboring unstressed syllable. In addition, lexical stress can also be used to distinguish a compound word 'a hotdog' from a noun phrase 'a hot dog'. Variation in the linguistic functions of F_0 _may account for perceptual difficulty typically experienced among adult native speakers of a non-tone language when consciously perceiving and distinguishing among lexical tones differing in pitch level or pitch contours. The aim of the present ERP study was to investigate whether the processing of lexical tones is affected by the listener's native language (tone or non-tone) even when the participants are not paying conscious attention to the stimuli, and whether such non-attentive perception can be altered by laboratory training, even in adults.

Previous behavioral studies have shown that native speakers of a non-tone language (e.g. English) poorly discriminate among lexical tones as compared with native speakers of a tone language (e.g. Mandarin Chinese), even when the latter are unfamiliar with the tones being tested [1-5]. This perceptual difficulty for speakers of non-tone languages is due in part to differences in the way lexical tones are processed among native and nonnative listeners of tone languages. Native speakers of a non-tone language have been shown to focus more on the average F_0_, and F_0 _offset or onset values, whereas speakers of a tone language focus more on F_0 _contour [6-8]. Interestingly, previous behavioral studies have also shown that adult native speakers of a non-tone language may improve in their perception of lexical tones after exposure to the tones either in a natural or classroom setting, or during laboratory training [3,4,9,10]. Training also affects the brain areas involved in lexical tone processing. fMRI studies comparing brain activation during lexical tone perception after versus before training showed an increase in activation in the left posterior superior gyrus [11,12]. In addition, right hemisphere activation was observed [12], especially in poor learners [11]. This suggests that the perceptual and neural systems involved in processing differences in pitch and pitch contours are still malleable, even in adulthood.

The discrimination or identification tasks used in the behavioral and fMRI studies on lexical tone perception involve conscious comparison or categorization. Performance in these experiments may therefore have been affected by factors such as working memory load or attention. In the present study we therefore studied the non-attentive discrimination of lexical tones and the effect of language background and training by using Event-Related brain Potentials (ERPs). ERPs can be recorded while the participant is presented with auditory stimuli, but engaged in an unrelated task such as watching a movie. The mismatch negativity (MMN) is a frontal negative ERP component occurring about 100–300 ms after stimulus onset. It is elicited by infrequent stimuli that deviate from frequently presented (standard) stimuli in pitch, duration, voice onset time, or other acoustic or phonetic properties [13]. Since this component is elicited even while people are asleep or in a coma, this component is regarded as an index of automatic processing of auditory differences, that is, processing that does not require voluntary attention. The MMN has been shown to increase in amplitude and, in some cases, to have a shorter peak latency as behavioral discrimination performance improves. In addition, changes in the MMN have been attested before changes in behavioral discrimination performance [14]. The MMN is therefore a useful tool to study the processing and acquisition of non-native language contrasts [14-20]. Since this technique taps into a different level of processing, and does not require overt attention and active comparison by the participant, this method may help us further tease apart the aspects of the stimuli that different language groups are differentially sensitive to at a non-attentive level of processing.

**Figure 1:**
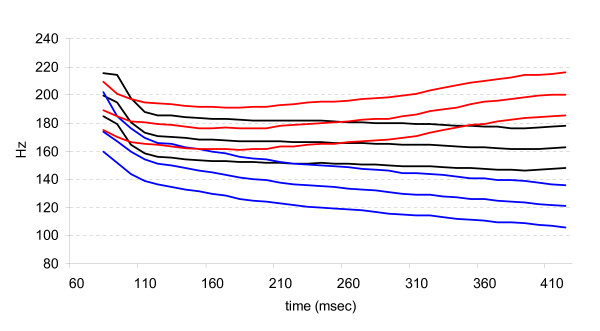
**Pitch tracks**. Pitch tracks of the three high-rising (red lines), mid-level (black lines) and low-falling (blue lines) tokens.

Only a few studies have employed the MMN to investigate the processing of lexical tones. Chandrasekaran et al. [21] investigated the effect of language background on lexical tone perception. Both Mandarin Chinese and untrained English speakers showed a MMN to tone contrasts in Mandarin Chinese. However, only the Chinese participants showed a larger MMN to a distinction that was acoustically more salient, suggesting that language background affects non-attentive processing of lexical tones to some extent. To investigate the effect of both training and language background, Kaan et al. [22] recorded ERPs from native speakers of English, Mandarin Chinese and Thai while they were presented with three Thai tones in an oddball paradigm. ERPs showed no differences between the groups before training. After a two-day perceptual training on the mid-level and low-falling tone, the English showed an increase in MMN amplitude to untrained high-rising deviants, whereas the Chinese showed a decrease in a later negativity in that condition. This suggested that native speakers of tone and non-tone languages were sensitive to different aspects of the stimuli as a result of training. However, no effect of training was observed on the (trained) low-falling tone deviant, to which all groups showed a large MMN before and after training. In addition, behavioral performance at the start of training was close to ceiling for all three subject groups. The differences found in the ERPs may therefore have not been indicative of improved perception of the tones. The ceiling performance may have been due to the use of only one token per tone condition, which did not encourage abstraction of contour categories. In the present experiment we therefore used multiple tokens of three Thai tones, all generated from one naturally produced token (see Methods and Figure 1).

Three subject groups (Thai, Mandarin Chinese and English speakers) were tested in an ERP oddball task in which they were presented with the stimuli while watching a silent movie. Although this task does not prevent participant from occasionally paying attention to the stimuli, the auditory stimuli are not task-relevant and do not require voluntary attention, in contrast to overt behavioral tasks. High-rising or low-falling tokens were presented as deviants among mid-level standard tokens, and vice versa. In addition a behavioral same/different discrimination task was conducted on the same stimuli. Both the behavioral discrimination and the ERP oddball task were conducted before and after a two-day perceptual categorization training task. We were particularly interested in seeing how the MMN and the later negativity for deviant versus standard stimuli would be affected by language background, training and the degree of behavioral improvement as a result of training. As one can see in Figure 1, the three tone categories differed from each other with respect to their F_0 _onset values, the steep F_0 _slope right after the F_0 _onset, as well as with respect to a later, more gradually developing F_0 _slope. Given that speakers of a non-tone language (English) have been shown to be sensitive to F_0 _onset and offset differences, whereas native speakers of a tone language are more sensitive to the later F_0 _contour, we expected the native English speakers to initially show a larger MMN than the native Chinese and Thai speakers. The Chinese and Thai speakers, on the other hand, were expected to show a more pronounced later negative effect, which may be related to the later contour differences [22]. As the native English speakers become more sensitive to the contour differences, we expected them to pattern more with the Thai and Chinese after training. Moreover, since the stimuli were meaningful words to Thai speakers, but not to Chinese and English speakers, we expected some differences related to the linguistic status of the stimuli. Linguistically perceived stimuli have been shown to involve the left hemisphere more than the right [3,18,23-26], but see [27,28]. The Thai were therefore expected to differ from the English and the Chinese participants in terms of the lateralization of the MMN and late negativity, at least, to the extent that the lateralization of scalp-recorded ERPs reflects hemispheric differences in the neural processes involved.

## Results

### Behavioral discrimination task and categorization training

Performance on the behavioral discrimination task (see Table 1) improved after training [F(1,32) = 20.64, p < 0.001]. This was more so for Chinese and English than for Thai participants, although the LANGUAGE GROUP by TEST TIME interaction did not reach significance [interaction: F(2, 32) = 2.87, p = 0.071; Post versus pre-training: English: t(11) = 3.46, p = 0.005; Chinese: t(11) = 4.13, p = 0.002; Thai: t(10) = 0.66, N.S.]. Before training, the three language groups differed from each other, with the English performing worse than the Chinese and Thai [LANGUAGE GROUP (pre-training): F(2,32) = 7.39, p < 0.001; English versus Chinese: p = 0.007; English versus Thai: p = 0.001]. The Chinese and Thai groups did not differ in their performance [p = 0.42]. After training, the groups did not differ in their ability to discriminate among the tones [LANGUAGE GROUP: F<1, N.S.].

**Table 1:** Results for the behavioral discrimination task

	English	Chinese	Thai
Pre-training	1.19 (0.66)	1.86 (0.39)	2.06 (0.65)
Post-training	2.06 (1.28)	2.57 (0.84)	2.21 (0.68)

The mean d' scores pre- and post-training per language group. Standard deviation in parentheses.

Performance in the categorization training (see Table 2) improved between the first and the last training sessions in all three language groups [F(1,31) = 33.92, p < 0.001]. The English showed the largest improvement, the Thai group the smallest [LANGUAGE GROUP by TEST TIME F(2,31) = 4.98, p = 0.013]. Overall, the English performed the worst [LANGUAGE GROUP: F(2,31) = 9.93, p < 0.001; English versus Chinese: p = 0.004; English versus Thai: p = 0.001; Thai versus Chinese: p = 0.26]. After the first training session, the language groups performed significantly different from each other [F(2,31) = 10.18, p < 0.001], with the English performing worse that the Chinese [p = 0.005] and the Thai [p < 0.001]. The Chinese and Thai did not differ significantly from each other [p = 0.19]. A similar statistical pattern was obtained after the last training session [LANGUAGE GROUP: F(2,32) = 7.18, p = 0.003; English versus Chinese: p = 0.007; English versus Thai: p = 0.001; Chinese versus Thai: p = 0.451].

**Table 2:** Results for the categorization training

	English	Chinese	Thai
First session	24.28 (14.28)	11.67 (7.39)	5.94 (5.59)
Last session	10.67 (7.36)	4.50 (3.65)	2.81 (3.86)

Mean percentage of errors after the first and last 30-minute training session, per language group. Standard deviation in parentheses.

Pre-and post training performance in the behavioral discrimination task correlated strongly with accuracy in the first and last categorization training, respectively [Pre-training: Pearson's ρ = -0.67, p < 0.001; Post-training: ρ = -0.63, p < 0.001]: the fewer errors made in the categorization training, the higher the d' scores in the discrimination task. This indicates that the behavioral discrimination task is a good measure of a participant's pre- and post-training perception ability.

### ERP experiment: movie comprehension questions

Mean comprehension accuracy on the movie-related questions in the ERP experiment was 84% (SD 7%), before as well as after training. Before training, the English group scored 87% correct (SD 5%), the Chinese 83% (SD 6%) and the Thai 81% (SD 9%). After training, the accuracy was 85% (SD 6%) for the English group, 85% (SD 9%) for the Chinese, and 84% (SD 7%) for the Thai groups. There were no significant differences in accuracy between pre- and post training sessions and/or among the language groups [ps > 0.2].

### ERPs to low-falling tones

#### MMN

**Figure 2:**
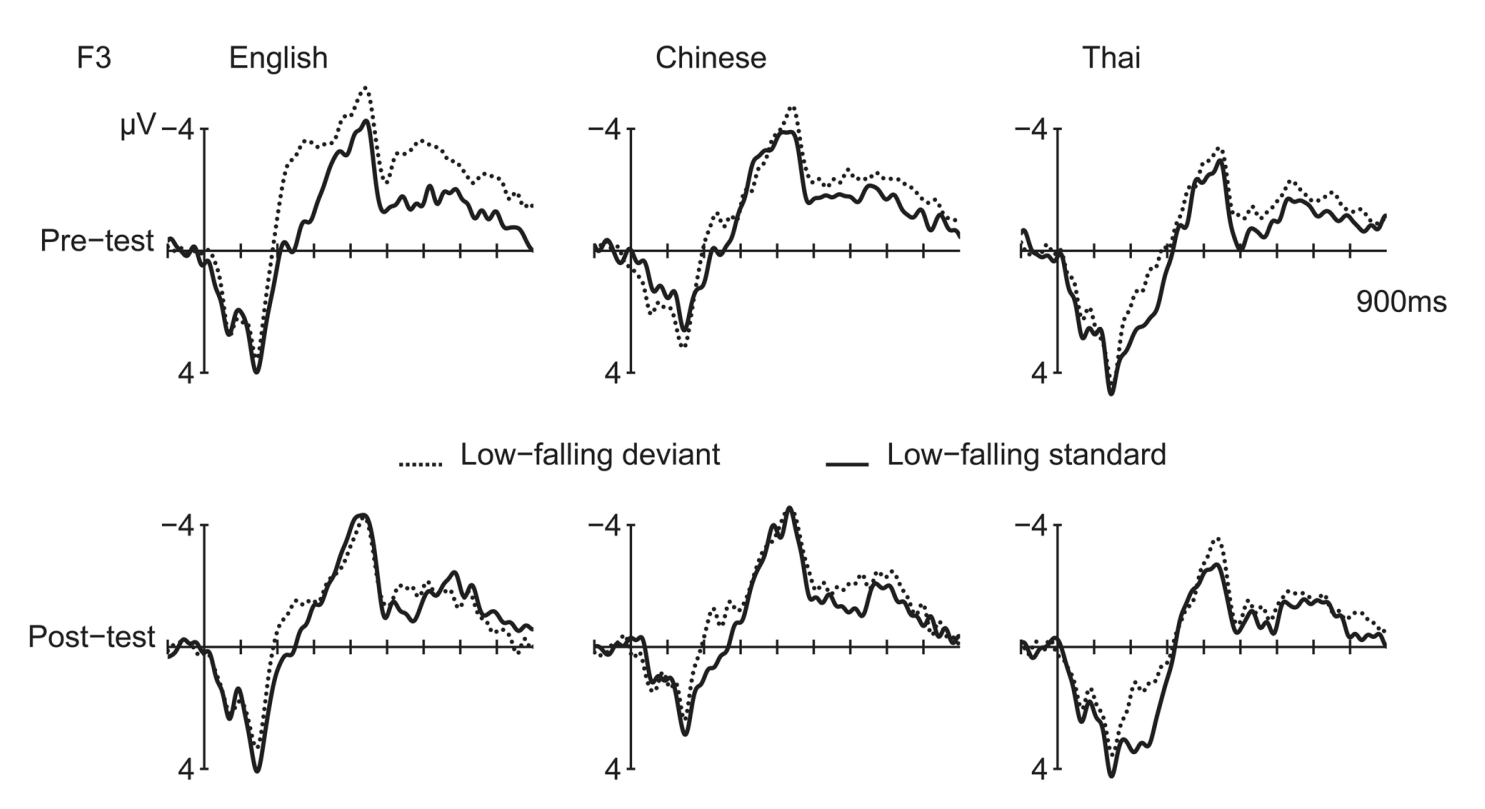
**ERPs to Low-falling deviants and standards**. ERPs at the left frontal electrode (F3) for the low-falling deviants (dotted line) versus standards (solid line).

**Figure 3:**
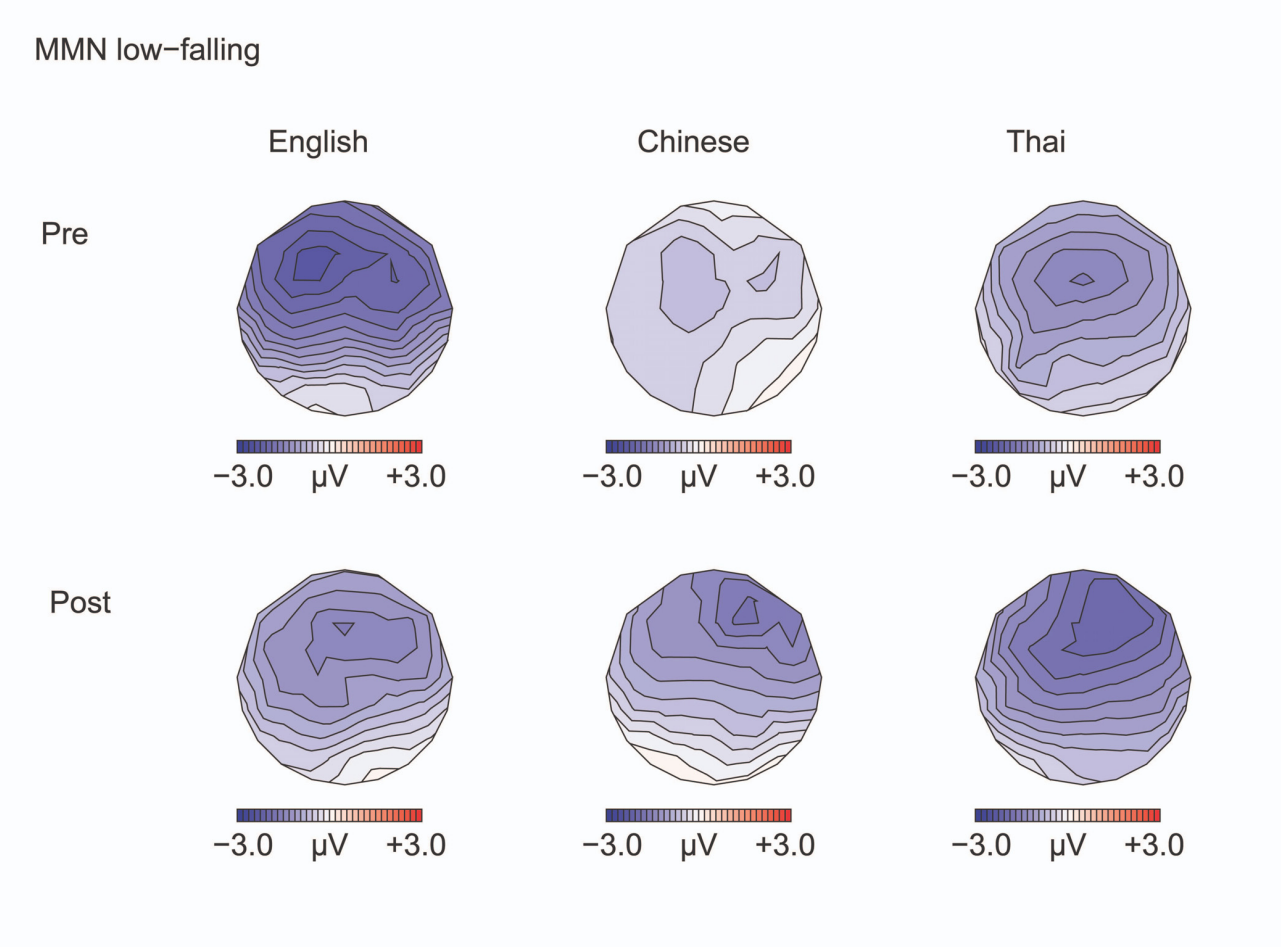
**Isovoltage maps to Low-falling deviants minus standards: MMN**. Isovoltage maps for the 100 ms window surrounding the most negative peak between 100–350 ms, for the low-falling deviants minus standards, defined separately for the language groups and test time.

The low-falling deviants (minus low-falling standards) showed a MMN at the F3 and F4 electrodes. ERPs for the F3 electrode are displayed in Figure 2. Figure 3 shows the isovoltage maps for the MMN. T-tests of the MMN amplitude at F3 and F4 versus a hypothetical zero showed that the MMN was significant before and after training in the English and Thai speakers [ps <0.004]. In the Chinese, the MMN was weakly present before training [p = 0.067] and significantly after [p < 0.001].

An ANOVA on the MMN amplitude (deviant minus standard) at the F3 and F4 electrodes showed an interaction of TEST TIME by HEMISPHERE [F(1,32) = 4.33, p = 0.046]: Before training, the MMN was numerically larger at the left hemisphere electrode [F3: -1.64 μV; F4: -1.49 μV ; t(34) = 0.78, N.S.]; after training, the MMN was numerically larger at the right electrode, with the difference almost reaching significance [F3: -1.54 μV; F4: -1.91 μV; t(34) = 1.97, p = 0.057]. Training also weakly affected the differences in the MMN between the groups [TEST TIME by LANGUAGE GROUP: F(2,32) = 2.69, p = 0.084]: The groups weakly differed from each other before training [F(1,32) = 2.89, p = 0.07], with the MMN being larger for the English compared to the Chinese [LSD post hoc comparison, p = 0.025], but not compared to the Thai [p = 0.12]. After training, the groups did not differ [ps > 0.68] (see Appendix).

The difference in MMN latency and amplitude after versus before training correlated weakly with the degree of learning: the greater the improvement in the behavioral discrimination task (d' scores post minus pre-training), the *earlier *the MMN peak and the *smaller *(i.e. less negative) the MMN amplitude was after compared to before training [Latency: Pearson's ρ = -0.32, p = 0.063; Amplitude: ρ = 0.32, p = 0.063].

#### Late negativity

**Figure 4:**
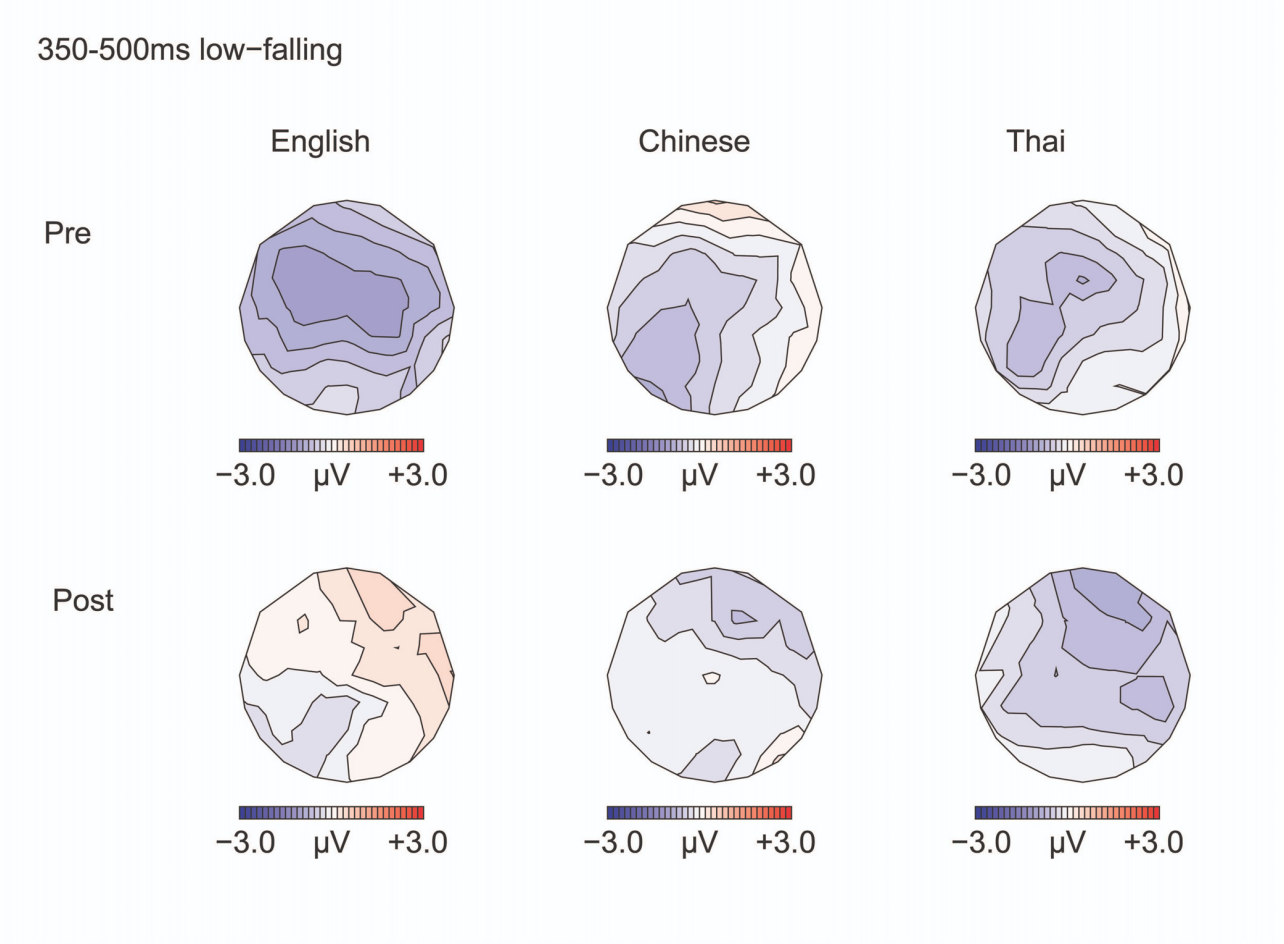
**Isovoltage maps to Low-falling deviants minus standards: 350–500 ms**. Isovoltage maps for the 350–500 ms window for the low-falling deviants minus standards.

A late negativity was seen for the deviant versus standard in the 350–500 ms window [Midline: F(1,32) = 7.29, p = 0.011; Lateral electrodes: F(1,32) = 6.75, p = 0.014] (see Figure 4.) The negativity was larger over the left hemisphere [F(1,32) = 9.18, p = 0.005], especially before training [CONDITION by TEST TIME by HEMISPHERE: F(1,32) = 6.95, p = 0.013; Pre-training: CONDITION by HEMISPHERE: F(1,32) = 13.94, p = 0.001; CONDITION: Left hemisphere: F(1,32) = 7.76, p = 0.009; Right hemisphere: F(1,32) = 1.32, p = 0.26; Post-training: no effects]. The degree of learning affected the change in the late negativity in the 350–500 ms interval: the larger the increase in d' scores from pre- to post-training, the *smaller *the late negativity in the 350–500 ms interval post- versus pre-training [Pearson's ρ = 3.68, p = 0.029].

**Figure 5:**
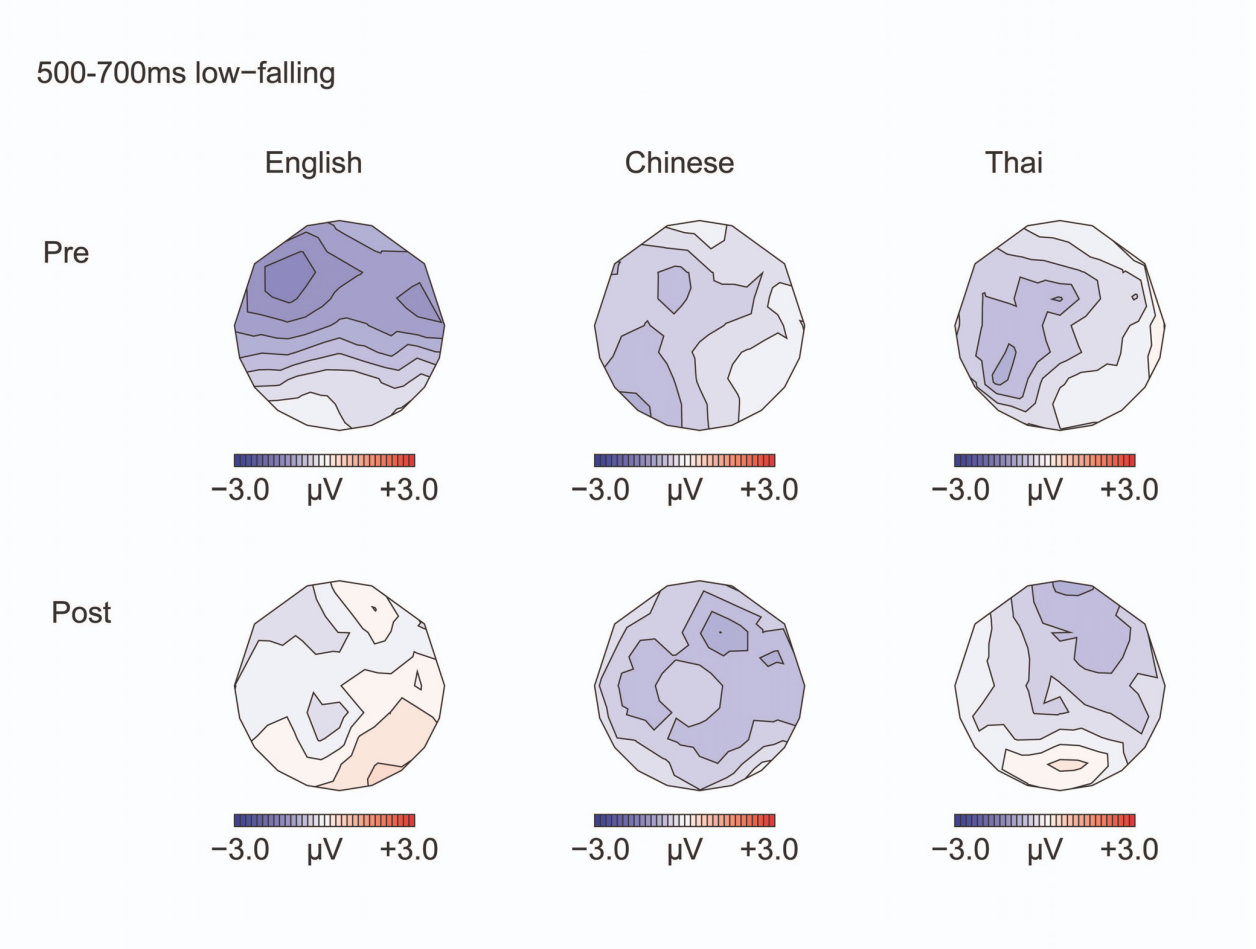
**Isovoltage maps to Low-falling deviants minus standards: 500–700 ms**. Isovoltage maps for the 500–700 ms window for the low-falling deviants minus standards.

The negativity persisted in the 500–700 ms interval, see Figure 5 [Midline: F(1,32) = 10.11, p = 0.003; Lateral: F(1,32) = 12.70, p = 0.001], and remained larger over the left than the right hemisphere [CONDITION by HEMISPHERE: F(1,32) = 5.203, p = 0.029; Effect of CONDITION: Left hemisphere: F(1,32) = 4.201, p = 0.049; Right: F(1,32) = 2.37, p = 0.13]. Effects involving the factor TEST TIME were not significant in this time window. The later negativity was not affected by language background in either the 350–500 ms or the 500–700 ms time window.

### ERPs to high-rising tones

**Figure 6:**
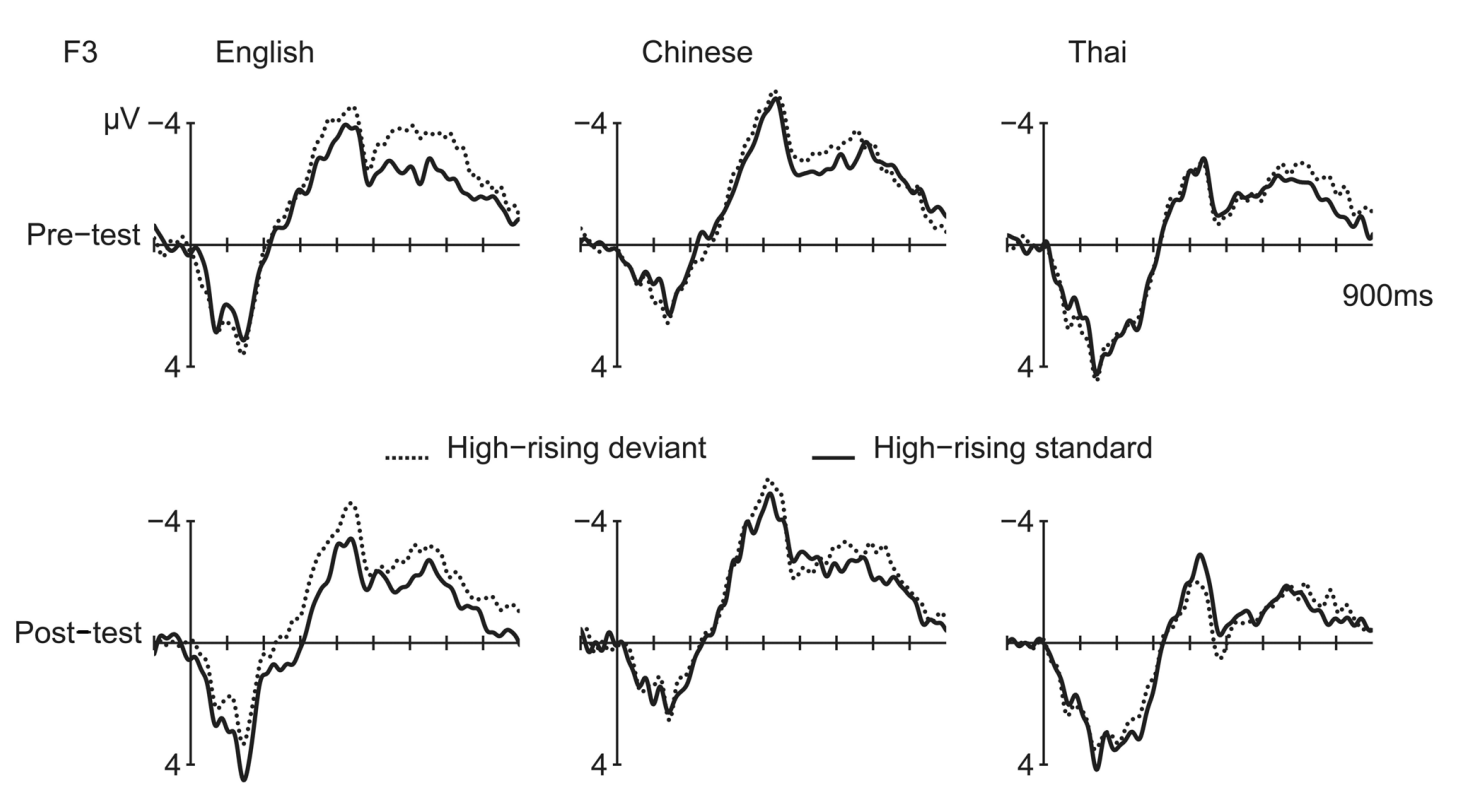
**ERPs to High-rising deviants and standards**. ERPs at the left frontal electrode (F3) for the high-rising deviants (dotted line) versus standards (solid line).

**Figure 7:**
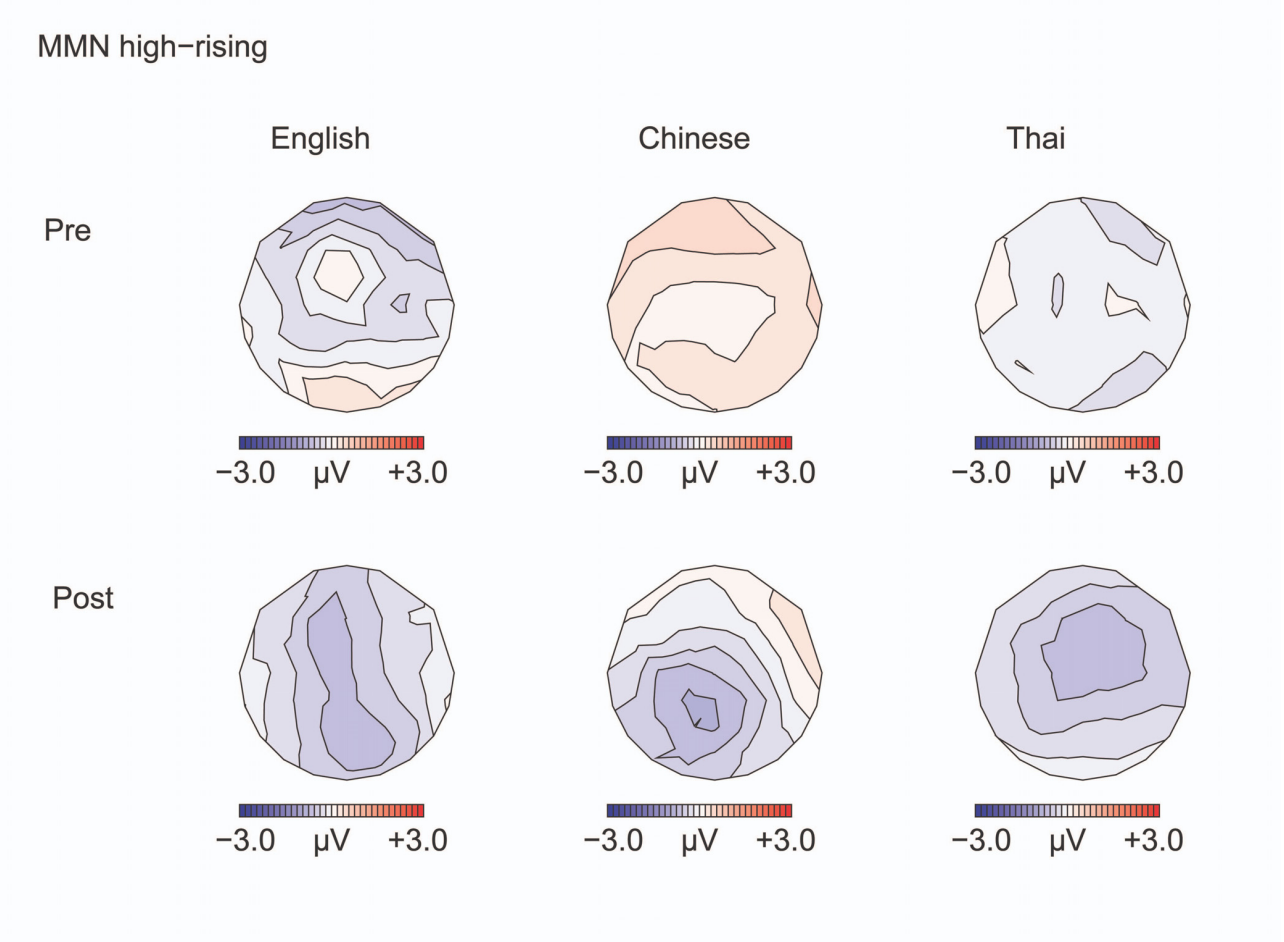
**Isovoltage maps to High-rising deviants minus standards:MMN**. Isovoltage maps for the 100 ms window surrounding the most negative peak between 100–350 ms, for the high-rising deviants minus standards, defined separately for the language groups and test time.

**Figure 8:**
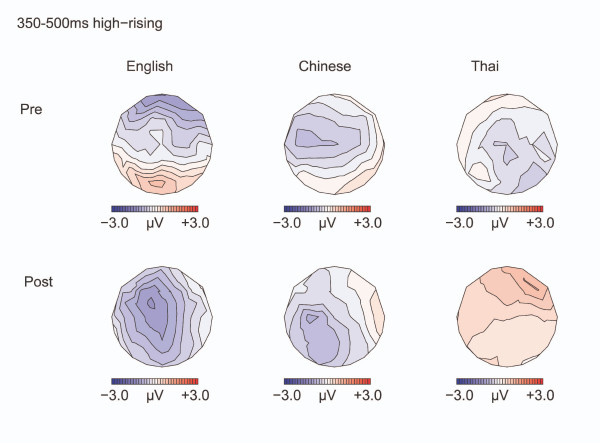
**Isovoltage maps to High-rising deviants minus standards: 350–500 ms**. Isovoltage maps for the 350–500 ms window for the high-rising deviants minus standards.

**Figure 9:**
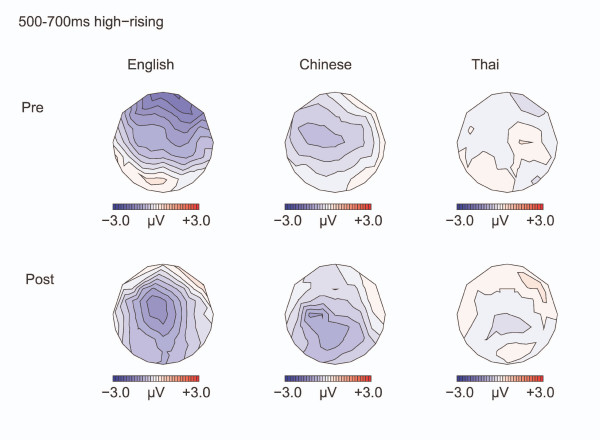
**Isovoltage maps to High-rising deviants minus standards: 500–700 ms**. Isovoltage maps for the 500–700 ms window for the high-rising deviants minus standards.

Results for the high-rising deviants versus high-rising standards are displayed in Figures 6 to 9.

#### MMN

Separate T-tests on the MMN amplitude (high-rising deviant minus high-rising standard) at F3 and F4 versus a hypothetical zero showed no significant differences in any of the groups before training [ps >0.18]. After training, the MMN was most robust in the native Thai speakers [Thai: p = 0.014; Chinese and English: ps >0.062]. ANOVAs showed no effects of the experimental manipulations on the MMN (amplitude or latency).

#### Late negativity

Between 350 and 500 ms, a negativity was elicited by the high-rising deviants versus standards, particularly over the left hemisphere [CONDITION by HEMISPHERE F(1,32) = 10.95, p = 0.002], see Figure 8. This left-lateralized negativity was only seen in the English and in the Chinese, but not in the Thai, leading to a weak interaction of CONDITION by HEMISPHERE by LANGUAGE GROUP [3-way interaction: F(2, 32) = 2.93, p = 0.068; CONDITION by HEMISPHERE, English: [F(1,11) = 6.37, p = 0.028. Chinese: F(1,11) = 9.04, p = 0.012; Thai: F(1,10)< 1, N.S.]. Training had an effect on the anterior-posterior distribution of the negativity [TEST TIME by CONDITION by ANTERIORITY F(4, 128) = 5.18, p = 0.018]: Pre-training, the negativity was numerically largest at frontal sites [CONDITION by ANTERIORITY: F(4, 128) = 3.96, p = 0.038], after training the negativity became broader in distribution and the two-way interaction between CONDITION and ANTERIORITY was no longer significant [F(4, 128) = 1.37, p = 0.26, N.S.]. Figure 8 suggests that this effect was mainly driven by the English group, however the interaction with LANGUAGE GROUP was not significant.

The negativity for the high-rising deviants persisted in the 500–700 ms interval (see Figure 9) [Midline: F(1,32) = 7.25, p = 0.011; Lateral: F(1,32) = 3.24, p = 0.066], with the negativity being larger over the left hemisphere [CONDITION by HEMISPHERE: F(1,32) = 13.619, p = 0.001; Effect of CONDITION: Left hemisphere: F(1,32) = 4.20, p = 0.049; Right hemisphere: F(1,32) = 2.73, p = 0.133]. The negativity was greater left than right at all except parietal regions [CONDITION by HEMISPHERE by ANTERIORITY: F(4,128) = 3.14, p = 0.032; CONDITION by HEMISPHERE was significant in all regions (ps ranging from 0.001 to 0.016) but for the parietal region (p = 0.15)]. The left lateralization was especially seen in the Chinese and English participants compared with the Thai [CONDITION by HEMISPHERE by LANGUAGE GROUP: F(2,64) = 3.43, p = 0.045; CONDITION by HEMISPHERE: Chinese: F(1, 11) = 8.88, p = 0.013 ; English: F(1, 11) = 9.172 p = 0.011; Thai: F <1, N.S. ]. As in the previous interval, the distribution was frontal before training and became broader after training [TEST TIME by CONDITION by ANTERIORITY: Midline: F(4,128) = 5.34, p = 0.014; Lateral: F(4,128) = 9.02, p = 0.002; CONDITION by ANTERIORITY, Pre-training: Lateral: F(4,128) = 4.66, p = 0.024; Post training: Lateral: F(4,128) = 2.44, p = 0.120. Effect of CONDITION Pre-training: Lateral frontal sites F(1,32) = 3.86, p = 0.058; Lateral: fronto-central sites: F(1,32) = 3.06, p = 0.09; Remaining regions: *p*s>0.18].

### Summary

All groups showed a MMN before and after training to the low-falling deviants. The MMN was larger over the right hemisphere after training. The English group tended to show a larger MMN before training than the Chinese, even though they performed worse in the behavioral tasks. Both MMN amplitude and latency *decreased *after training the more the participant improved in the behavioral discrimination task. The MMN was followed by a slow negativity, which was slightly larger over the left than the right hemisphere, and reduced in amplitude as a function of learning. The high-rising deviants elicited no or only a small MMN. The late negativity in this condition was left-lateralized for the English and the Chinese groups. The later negativity was frontal before training, but became broader after training.

## Discussion

The aim of the present ERP study was to investigate the processing of lexical tones when participants are not forced to pay attention to the stimuli, as opposed to previous studies using behavioral techniques only, and to see to what extent such non-attentive processing is affected by training and by native language background. In contrast to previous ERP studies [21,22], we used multiple tokens per stimulus type to encourage the formation of abstract contour categories and to avoid pre-training ceiling effects. Results from the behavioral discrimination task suggest that this manipulation was successful: performance significantly increased after training in the English and the Chinese groups, who were initially unfamiliar with the Thai stimuli used. Furthermore, behavioral discrimination scores correlated significantly with performance in the categorization training task.

Based on previous experiments showing that native speakers of a non-tone language are more sensitive to F_0 _onset and offset when discriminating lexical tones [6-8,22], we predicted that the English group would show a larger MMN to the deviant categories; the Chinese and Thai on the other hand, previously shown to be more sensitive to F_0 _contours, were expected to show a more robust later effect. In addition, given that the stimuli were meaningful words in Thai, we predicted a lateralization difference between Thai on the one hand, and English and Chinese on the other.

Our predictions were only partly borne out. We will discuss our findings in turn for the MMN and the late negativity.

### The MMN

All groups showed a MMN to the low-falling tone deviants, before as well as after training; whereas no, or only a smaller MMN was elicited by the high-rising tone deviants. Note that two of the three low-falling tones have an onset frequency falling below the range of the mid-level tones (see Figure 1). The onset frequency of the high-rising tones, on the other hand, falls within the range of that of the mid-level tokens. It is therefore likely that the large MMN found for the low-falling tones reflects differences in F_0 _onset between the deviant and standard stimuli presented in the same block. These differences were much smaller in the high-rising tones [29,30].

The MMN was weakly affected by native language background: the English showed a larger MMN to the low-falling tones than the Chinese before training. This supports previous findings [6-8,22] that speakers of a non-tone language are more sensitive to differences in onset F_0_. Our English speaking participants may have been more sensitive to the early F_0 _differences in the Low-falling conditions, eliciting a larger MMN compared to the Chinese and Thai groups. Note that although the English language group showed the largest MMN before training, they performed worse than the Thai and Chinese in the behavioral discrimination and training. This can also be accounted for by the different sensitivity of tone versus non-tone language speakers. The behavioral tasks probed participant's sensitivity to differences in F_0 _slope and direction rather than F_0 _onset, and was therefore harder for non-tone language speakers. The categorization training with multiple tokens per type caused the English speaking participants to become more sensitive to the direction of the pitch contour. This may have induced a modulation of their non-attentive perception, hence a reduction of the MMN amplitude in the English language group after training to the level of the speakers of tone languages.

The MMN became smaller and earlier with behavioral improvement. Typically, the MMN has been found to become larger after training [15,16,18]. The decrease in MMN amplitude therefore suggests that the participants, and especially the learners, non-attentively perceived the stimuli in a different way and became less sensitive to the F_0 _onset differences after training, or at least, as a result of repeated exposure.

For all three language groups, the MMN to the low-falling deviants became more prominent over the right hemisphere after training. This is in contrast to several previous ERP studies that reported an increase in MMN over the left hemisphere after training on linguistic contrasts [18]. To the extent that the lateralization of scalp-recorded ERPs reflects hemispheric differences in the neural processes involved, our findings suggest that even native speakers of Thai employ the right hemisphere more than the left in processing the low-falling versus mid-level tone contrast. This is in spite of the fact that the stimuli are meaningful words for the Thai. A previous study on Mandarin Chinese speakers reports a similar right hemisphere distribution for meaningful lexical tone contrasts [28]. Under an alternative account of hemispheric specialization of speech, the left hemisphere is involved in processing rapid formant transitions, whereas the right hemisphere deals with slower differences in pitch [31]. It may therefore be the case that our participants became more sensitive to the gradual change in F_0 _contour, focused less on the differences in F_0_onset values and the abrupt change in F_0 _at the beginning of the stimuli, and thus involved the right hemisphere more as a result of training.

### The later negativity

Second, we were interested in the later negativity. In contrast to our prediction, no difference was seen between English and Chinese speakers. All groups displayed a negativity to both the low-falling and high-rising deviants versus standards. Late negativities reported in the literature have been associated with cognitive, possibly non-attentive processing of sound change [32], or processing at a higher level of abstraction [33,34] including harmonic integration in music contexts [35,36]. Alternatively, the late negativity may reflect reorienting of attention after involuntary attention to deviant stimuli [37,38]. A smaller late negativity may then indicate a more efficient neural processing, or less attentional reorienting. For the low-falling deviants, the late negativity became less left-lateralized after training and smaller in amplitude the more the participant improved on the behavioral task. For the high-rising deviants, the Chinese and English speaking groups showed a left-lateralization of this negativity for the high-rising deviants, regardless of training.

Note that the low-falling stimuli continue to differ from the mid-level stimuli in terms of a falling pitch slope right after the initial sharp fall in F_0 _(see Figure 1). The high-rising tones, on the other hand, only show a gradual increase in F_0 _compared to the mid-level tones, starting at around 290 ms after onset. Two of the three high-rising tokens start to exceed the F_0 _range of mid-level tones even later. The contour deviance is therefore more subtle in the high-rising than low-falling conditions in the current study. Since training focused on contour differences, the processing of the low-falling contour may therefore have required less effort after training in the learners, hence the reduction of the late negativity in this condition, but not in the high-rising condition in this experiment. The left-lateralization of the late negativity in the high-rising condition in the Chinese and English groups suggests that the non-native language groups process the Thai high-rising contour in a manner that is different from native Thai speakers. Comparable to the MMN, and the late negativity in the low-falling condition, this waveform may shift from the left to the right hemisphere when listeners become more proficient in detecting the contours. Apparently, the categorization training was not sufficient to give rise to these effects in the non-native speakers. It remains to be seen if a longer period of training will lead to a shift in hemispheric lateralization to be observed.

### The relation between ERPs and behavioral data

Behavioral studies on the perception and acquisition of foreign language contrasts are potentially confounded by the attentional and memory load that is imposed by most discrimination or categorization tasks. Using ERPs overcomes this problem because passive listening tasks can be used which do not require any explicit attention or overt behavioral response from the participant. On the other hand, behavioral and ERP studies may tap into different aspects of processing. ERPs may be more sensitive to differences in physical properties of the stimuli than behavioral tasks. In addition, behavioral studies may encourage participants to actively form abstract perceptual categories, whereas passive listening oddball tasks, as used in the current ERP study, may do so to a lesser extent. It is therefore not surprising that we observed some discrepancies between our behavioral and ERP data. ERPs are therefore a good complementary method to behavioral studies, and are a good tool to help uncover what aspects of the stimuli different language groups are differently sensitive to.

We have already discussed the larger MMN to low-falling deviants seen in the English group pre-training in spite of this group's poor performance on the behavioral tasks. This can be accounted for by the MMN being a reflection of a participant's sensitivity to early differences between the stimuli, whereas the behavioral tasks tapped more into the participant's ability to actively form categories on the basis of the later pitch contour. In contrast to the MMN, the late negativity did not correspond to the behavioral differences observed between the groups before training. However, the amplitude of the late negativity in the low-falling conditions did correlate with behavioral improvement: the late negativity amplitude became smaller the more the participant improved in the discrimination task. In the high-rising condition, the late negativity became more broadly distributed after training. Finally we would like to point out that in spite of differences in language background, all participant groups elicited largely similar ERP components and that, with the exception of the MMN, the effect of training was largely the same among the groups. This suggests that the neural mechanisms involved in non-attentively perceiving tone stimuli and the effects of training thereon may have been largely unaffected by language background.

## Conclusion

In sum, native speakers of English, Chinese and Thai recruited largely similar neural mechanisms when non-attentively processing Thai lexical tones. Training induced comparable changes in the language groups. However, and converging with results from behavioral methods using different stimuli and techniques, we found that native speakers of English were initially more sensitive to early F_0 _differences before training. After training, this language group became more similar to native tone-language speakers. In addition, native speakers of English and of Mandarin Chinese processed the late shallow contour in the high-rising Thai tone differently from native Thai speakers. Future experiments will determine whether this can be affected by a more extended period of training.

## Methods

### Participants

Twelve native speakers of American English (8 men), 12 native speakers of Mandarin Chinese (People's Republic of China) (6 men), and 11 native speakers of Thai (5 men) were recruited from the University of Florida community. Informed consent was obtained from each participant according to the procedures of the University of Florida Institutional Review Board. All participants were healthy young adults, aged 19–35, right handed as assessed by the Edinburgh handedness inventory [39], and with no history of neurological disease or language disorders as indicated by a self-report. All had a minimal bilateral hearing range of 500 to 8,000 Hz measured at 25 dB HL. The American English speakers did not have any experience with a tone language; the native Chinese speakers did not have experience with any other tone language, except one who spoke a Chinese dialect in addition to Mandarin Chinese. Participants were paid for participation. Ten additional participants were run, but were omitted from analysis because of incomplete data sets (due to technical difficulties or failure to return for all sessions).

### Stimuli

Nine stimuli were synthesized on the basis of one naturally generated instance of the Thai mid-level tone syllable [k^h^a:] produced by a female native speaker of Thai and digitized at 22050 Hz sampling rate with a 16-bit amplitude resolution. Using the Praat speech analysis software, the original mid-level tone was shortened from 610 ms to 450 ms. The pitch contour of this mid-level tone was then manually changed to approximate the pitch contours of the natural tokens of the Thai low-falling and high-rising tones. The entire F_0 _contour of each of the three resulting stimuli was then shifted down -15 Hz and -30 Hz to simulate three different talkers, thus yielding three tokens for each of the three tone types, see Figure 1. All stimuli were normalized for RMS amplitude (98% of the scale). All 3 tokens of each tone were then presented to two native Thai speakers (one male and one female) and were judged to be acceptable exemplars of each of the three tone categories. Sound files and spectrograms of each token are provided as supplementary materials (Additional files 1, 2, 3, 4, 5, 6, 7, 8, 9, 10, 11, 12, 13, 14, 15, 16, 17, 18).

### Procedure

Participants were tested on these stimuli on four consecutive days. Stimuli were presented binaurally, one at a time over head phones at a comfortable hearing level (65 dB). An ERP oddball task was conducted on Days 1 and 4; two categorization training sessions each were conducted on Days 2 and 3, with a behavioral discrimination task either preceding (Day2) or following (Day 3) the training.

### Behavioral discrimination task

In the behavioral discrimination task (Days 2 and 3) the participant heard a sequence of three different stimuli A B C, separated by 575 ms. A and B were always from the same tone category (either low-falling, high-rising or mid-level). The last stimulus, C, was either of the same or of a different contour category, and the participant was asked to indicate whether the contour was same or different by clicking a mouse button (113 trials total: 108 experimental trials and 5 warm-up trials that were not analyzed). The response side for the 'same' and 'different' responses was counterbalanced among participants. If no response was given after 3 seconds, the next trial started. Responses longer than 3 seconds (2.2–3.5% per session and language group) were treated as no-response errors. D' scores were calculated on the percentage of hits (correct 'different' response in case tone C was of a different type than A and B) and false alarms (incorrect 'different' response when A, B and C were of the same category). Null responses were not included in d'score calculation.

### Categorization training

In the categorization training sessions (Days 2 and 3), participants heard one stimulus per trial. They were asked to classify a token as being of tone type A, B or C by clicking a box on the screen [4,5,22]. During the introduction phase of the training, they heard the three tokens of tone type A (low-falling), followed by the three tokens of tone type B (mid-level), followed by the three tokens of tone type C (high-rising). After this was repeated three times, the tokens were presented in random order for a total of 81 trials (each token presented 9 times) and accuracy was recorded. Participants were allowed to replay the sound. If an incorrect response was given, the frame around the box with the correct answer would blink. The inter-trial interval was 3 seconds. Responses longer than 3 seconds (including replays) were omitted from analysis. This amounted to 0.6–3.2% of the data per session and language group. One session lasted 30 minutes and was repeated on the same day after a short break. Data from one Chinese participant for the first training session on Day 2 were missing due to technical failure. Hence, this participant is omitted in all analyses involving this first session.

### ERP oddball experiment

In the ERP oddball task (Days 1 and 4), the stimuli were presented in a continuous stream. Four stimulus blocks were presented, the order counterbalanced across participants: (1) mid-level presented as standard, high-rising as deviant; (2) high-rising as standard, mid-level as deviant; (3) mid-level as standard, low-falling as deviant; (4) low-falling as standard, mid-level as deviant. A total of 1200 stimuli were presented per block: 1080 of the standard category and 40 of each of the three deviant tokens (i.e., 10% deviants). The inter-stimulus (offset-to-onset) interval was randomized between 500–650 ms to prevent interference from regular biological rhythms on the waveforms. The order of the stimuli was pseudo randomized such that two deviants were separated by at least two standards. The length of each block was 17 minutes. While the auditory stimuli were presented over headphones, participants watched a silent movie (Charlie Chaplin's 'The Gold Rush' or Buster Keaton's 'The General'). They were told that they would receive questions about the movie after each of blocks, and were instructed to ignore the sounds. A different movie fragment was played during each session. Each ERP session lasted about 2 hours, including set up and debriefing.

EEG was recorded from 39 Ag/AgCl scalp electrodes mounted in an elastic cap with active shielding (Easy-Cap, Falk Minow, Herrsching-Breitbrunn, Germany) combined with an ANT amplifier (ANT Software b.v., Enschede, The Netherlands). Electrode positions used were: Midline: Fz, FCz, Cz, CPz, Pz; Lateral left/right hemisphere: FP1/2, F7/8, F5/6, F3/4, FT7/8, FC5/6, FC3/4, T7/8, C5/6, C3/4, TP7/8, CP5/6, CP3/4, P7/8, P5/6, P3/4, O1/2. Horizontal and vertical EOG were recorded from the outer canthi, and below and above the right eye, respectively. Additional electrodes were placed on the right and left mastoids. The signal was acquired using the left mastoid as reference, but was arithmetically re-referenced off-line to the mean of the left and right mastoids. Electrode impedance was kept below 5 KOhm. The signal was sampled at a rate of 512 Hz, and was filtered off-line between 0.3 and 30 Hz. We only analyzed low-falling and high-rising stimuli. These were always presented with mid-level stimuli in the presentation blocks. Any differences between the ERPs to the low-falling and high-rising tones can therefore not be due to different alternate stimuli in the presentation blocks. Epochs were defined spanning -100 to 900 ms from the stimulus onset. EEG to low-falling and high-rising tone deviants were averaged separately. We also separately averaged the EEG to 120 low-falling and high-rising tones when these were used as standards. To avoid any potentially confounding effects from preceding deviant tones, we selected 120 standard stimuli that were preceded and followed by a standard stimulus. Trials with eye movements and other artifacts were rejected. The percentage of rejection was on average 28% per condition (SD 15%) in the Chinese group; 20% in the English group (SD 9%), and 26% (SD 13%) per condition in the Thai group.

The *mismatch negativity *was analyzed using the F3 and F4 electrodes. These were electrodes where the MMN was largest on the lateral sites. First, difference waves (deviant minus standard) were calculated for the high-rising deviants minus standards, and low-falling deviants minus standards. Next, the most negative peak was found between 100 and 350 ms, and the mean amplitude for the windows spanning 100 ms centered around this peak was calculated for every channel, participant, tone type and session. Analyses were conducted on the mean difference in amplitude thus calculated and on the peak latency.

A *later negative component *was observed as well. Since we had no clear prediction as to the scalp distribution of this component and since the wave did not have a clear peak, analyses were conducted on the mean amplitudes to both the deviant and standard tones, and included a large number of electrodes. Statistical analyses were conducted on the mean amplitudes between 350–500 ms and 500–700 ms, based on visual inspection, using lateral (F3/4, F5/6, F7/8, FC3/4, FC5/6, FT7/8, C3/4, C5/6, T7/8, CP3/4, CP5/6, TP7/8, P3/4, P5/6, P7/8), as well as midline (Fz, FCz, Cz, CPz, Pz) electrodes.

ERP data were analyzed separately for low-falling and high-rising tones, using an (SPSS) General Linear Model multivariate repeated measures procedure with the within-participant factors: TEST TIME (pre/post training), and, when applicable, CONDITION (standard, deviant), HEMISPHERE (2 levels) and/or ANTERIORITY (5 levels). LANGUAGE GROUP was included as a between-participants factor (3 levels). When a two or three-way interaction was significant, separate analyses were conducted to determine the source of the interaction. For the late negativity only effects involving the factor Condition are reported below. When interactions involving factors with more than two levels were significant, F- and p-values were reported after the Greenhouse-Geisser correction to control for violations of sphericity [40].

## Abbreviations

EEG: electroencephalogram; ERP: event-related potential; F_0_: fundamental frequency; LSD: least significant difference; MMN: mismatch negativity; N.S.: not significant; SD: standard deviation.

## Authors' contributions

EK designed and coordinated the ERP study, analyzed the data and drafted the manuscript. CMB and MB carried out the experiment. RW conceived of the study, constructed the stimuli and designed the discrimination and training tasks. All authors read and approved the final manuscript.

## Appendix

Tests involving 34 lateral electrodes did not show any significant interaction of location with Language group except for a weak interaction of TESTTIME BY ANTERIORITY BY LANGUAGE for the low-falling deviants minus standards [F(8, 128) = 2.50, p = 0.076]. Numerically, the English group showed a larger frontal negativity than the Thai and Chinese before, but not after training. This effect is the same as the weak LANGUAGE BY TESTTIME interaction found for the F3 and F4 electrodes discussed in the main text.

## Supplementary Material

Additional File 1 Sound file of the first low-falling token. Click here for file

Additional File 2 Sound file of the second low-falling token. Click here for file

Additional File 3 Sound file of the third low-falling token. Click here for file

Additional File 4 Sound file of the first mid-level token. Click here for file

Additional File 5 Sound file of the second mid-level token. Click here for file

Additional File 6 Sound file of the third mid-level token. Click here for file

Additional File 7 Sound file of the first high-rising token. Click here for file

Additional File 8 Sound file of the second high-rising token. Click here for file

Additional File 9 Sound file of the third high-rising token. Click here for file

Additional File 10 Spectrogram of first low-falling token. Click here for file

Additional File 11 Spectrogram of second low-falling token. Click here for file

Additional File 12 Spectrogram of third low-falling token. Click here for file

Additional File 13 Spectrogram of first mid-level token. Click here for file

Additional File 14 Spectrogram of second mid-level token. Click here for file

Additional File 15 Spectrogram of third mid-level token. Click here for file

Additional File 16 Spectrogram of first high-rising token. Click here for file

Additional File 17 Spectrogram of second high-rising token. Click here for file

Additional File 18 Spectrogram of third high-rising token. Click here for file
