# Supplementary figures and images for: Thai lexical tone perception in native speakers of Thai, English and Mandarin Chinese: An event-related potentials training study
Source: BMC Neurosci. 2008 Jun 23;9:53. doi: 10.1186/1471-2202-9-53 (PMC2483720; doi:10.1186/1471-2202-9-53)

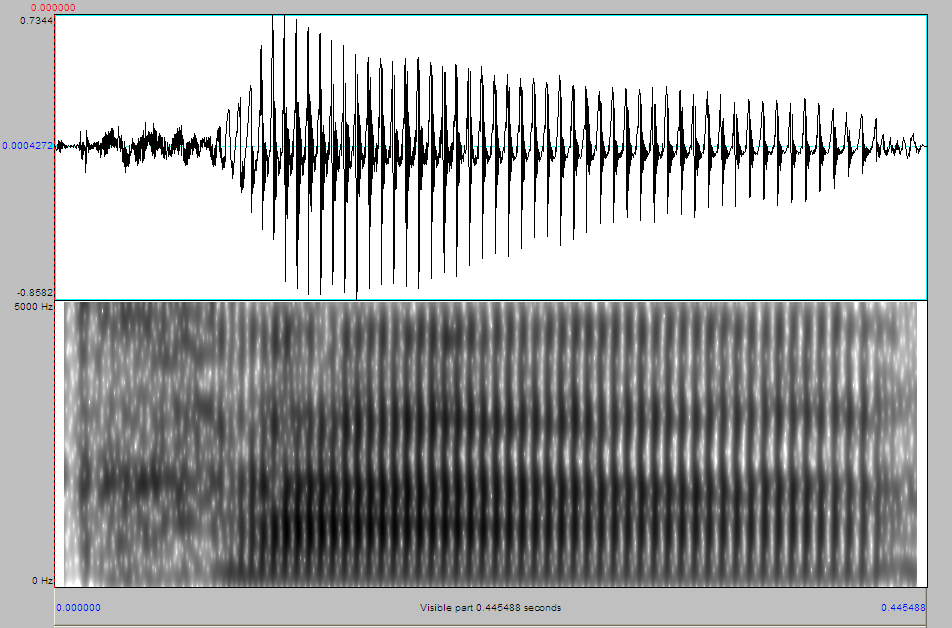

Supplement: Additional File 10 — Spectrogram of first low-falling token. [file 1471-2202-9-53-S10.bmp]

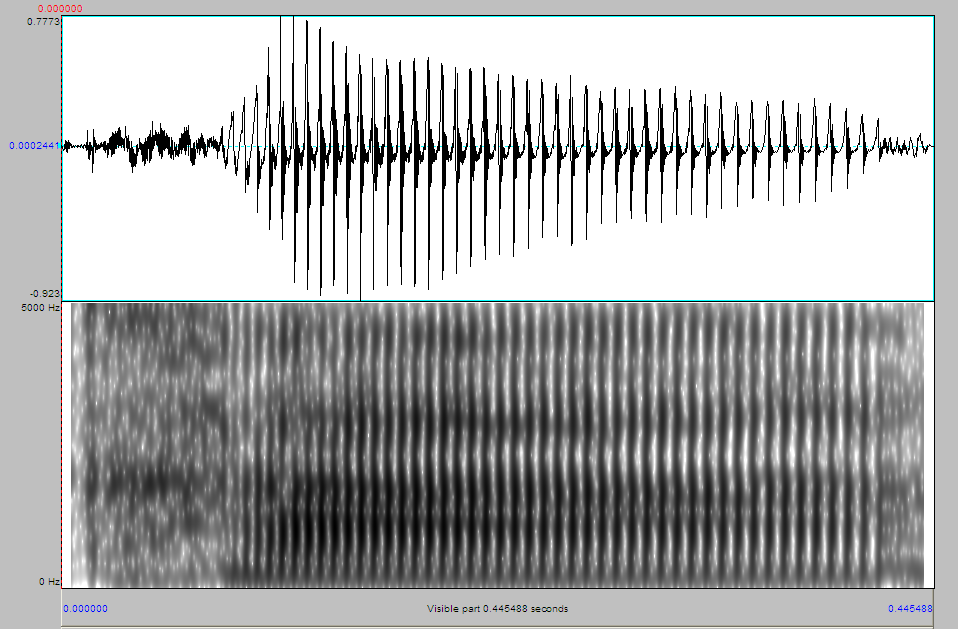

Supplement: Additional File 11 — Spectrogram of second low-falling token. [file 1471-2202-9-53-S11.bmp]

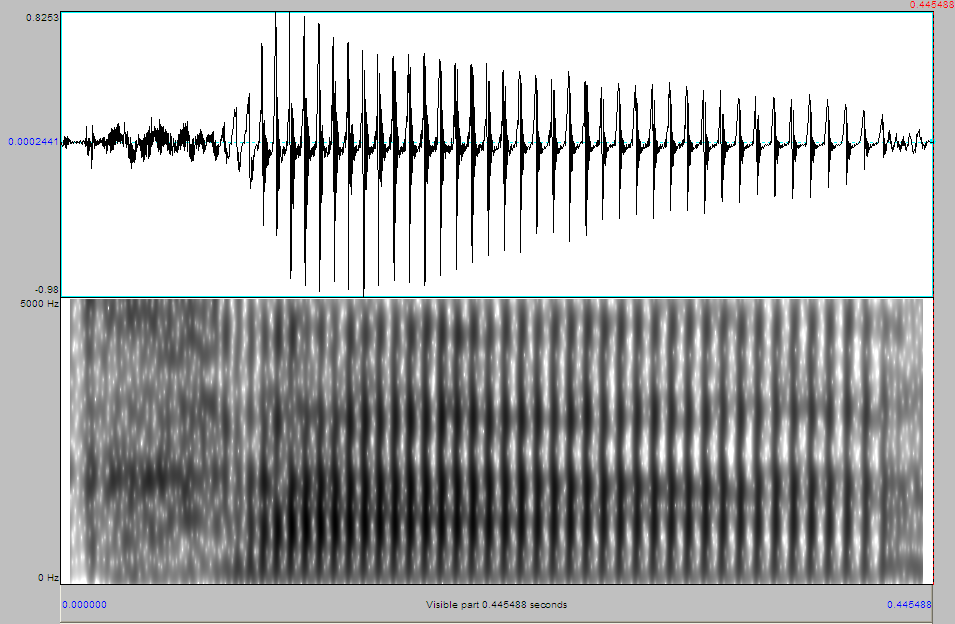

Supplement: Additional File 12 — Spectrogram of third low-falling token. [file 1471-2202-9-53-S12.bmp]

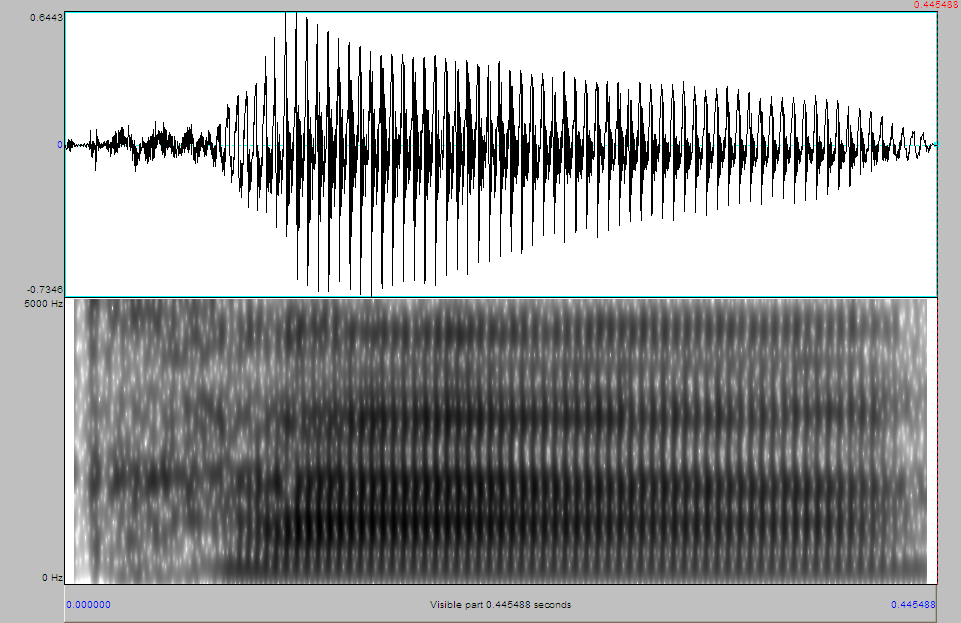

Supplement: Additional File 13 — Spectrogram of first mid-level token. [file 1471-2202-9-53-S13.bmp]

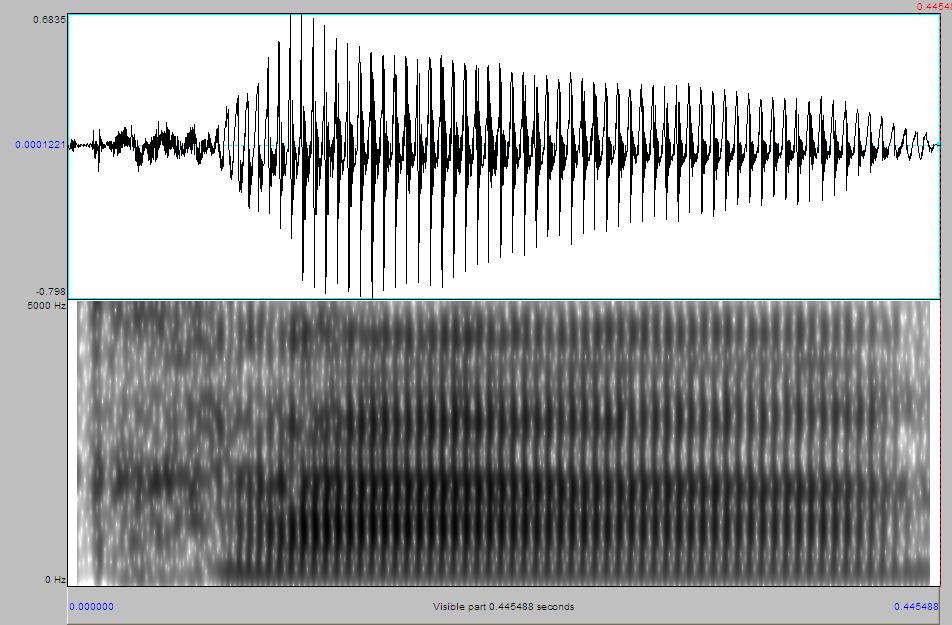

Supplement: Additional File 14 — Spectrogram of second mid-level token. [file 1471-2202-9-53-S14.bmp]

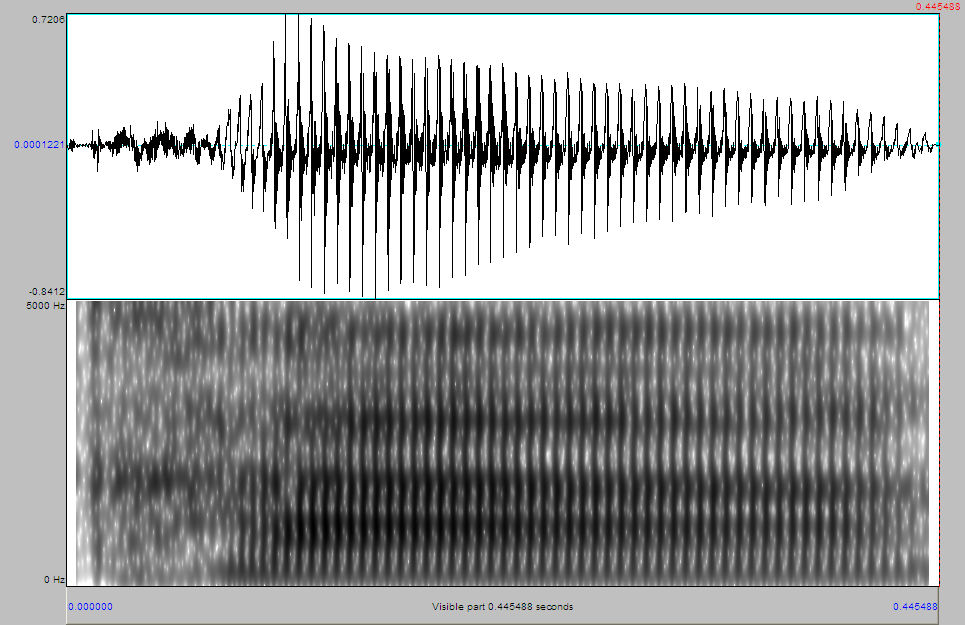

Supplement: Additional File 15 — Spectrogram of third mid-level token. [file 1471-2202-9-53-S15.bmp]

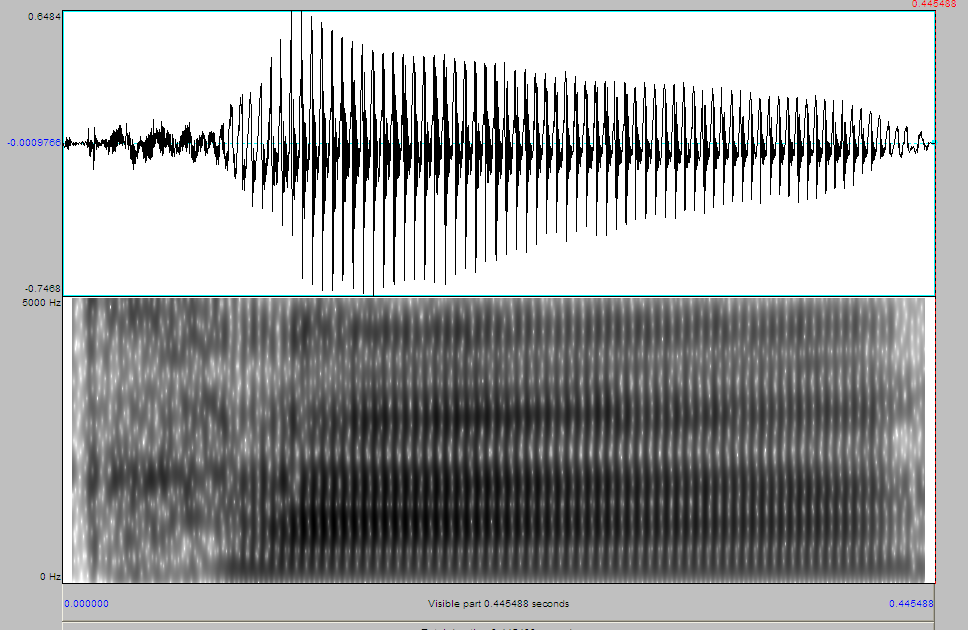

Supplement: Additional File 16 — Spectrogram of first high-rising token. [file 1471-2202-9-53-S16.bmp]

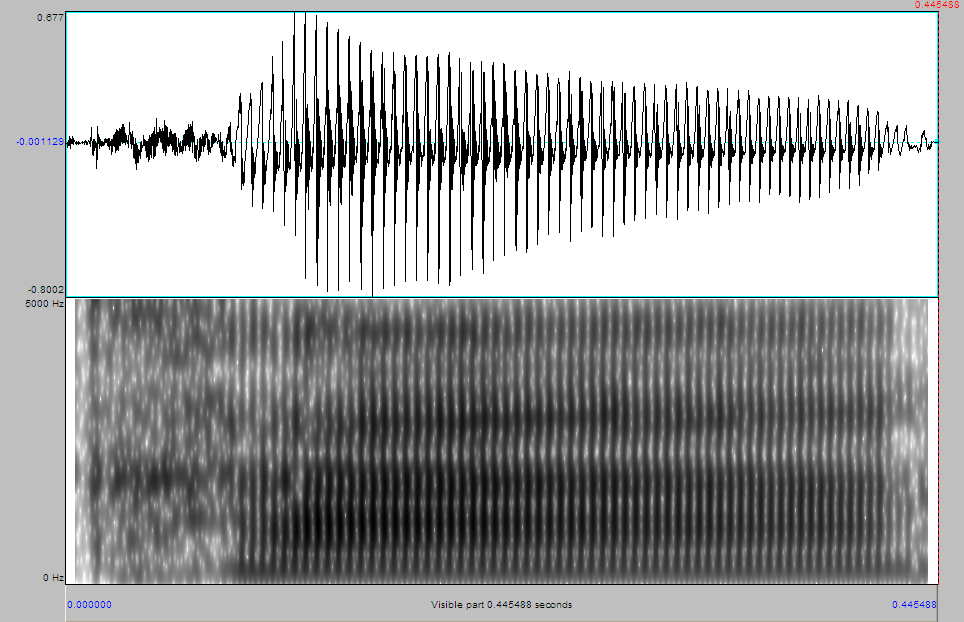

Supplement: Additional File 17 — Spectrogram of second high-rising token. [file 1471-2202-9-53-S17.bmp]

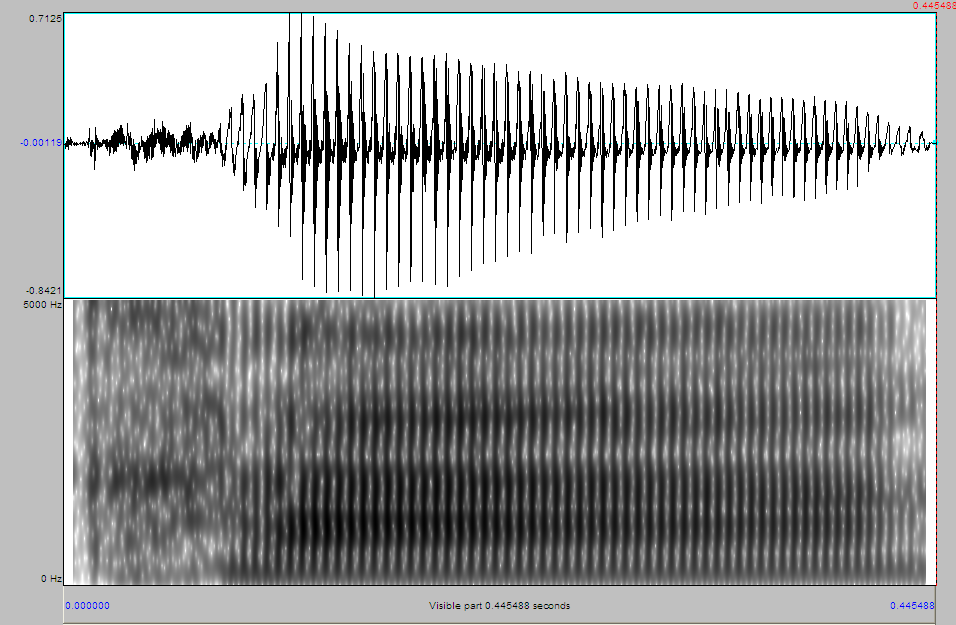

Supplement: Additional File 18 — Spectrogram of third high-rising token. [file 1471-2202-9-53-S18.bmp]
